# DNA from resin-embedded organisms: Past, present and future

**DOI:** 10.1371/journal.pone.0239521

**Published:** 2020-09-28

**Authors:** David Peris, Kathrin Janssen, H. Jonas Barthel, Gabriele Bierbaum, Xavier Delclòs, Enrique Peñalver, Mónica M. Solórzano-Kraemer, Bjarte H. Jordal, Jes Rust

**Affiliations:** 1 Section Paleontology, Institute of Geosciences, University of Bonn, Bonn, Germany; 2 Institute of Medical Microbiology, Immunology and Parasitology, Medical Faculty, University of Bonn, Bonn, Germany; 3 Department of Earth and Ocean Dynamics and Biodiversity Research Institute (IRBio), Faculty of Earth Sciences, Universitat de Barcelona, Barcelona, Spain; 4 Geological and Mining Institute of Spain (Geominero Museum), Valencia, Spain; 5 Department of Palaeontology and Historical Geology, Senckenberg Research Institute, Frankfurt am Main, Germany; 6 Museum of Natural History, University Museum of Bergen, University of Bergen, Bergen, Norway; University of Florence, ITALY

## Abstract

Past claims have been made for fossil DNA recovery from various organisms (bacteria, plants, insects and mammals, including humans) dating back in time from thousands to several million years BP. However, many of these recoveries, especially those described from million-year-old amber (fossil resin), have faced criticism as being the result of modern environmental contamination and for lack of reproducibility. Using modern genomic techniques, DNA can be obtained with confidence from a variety of substrates (e.g. bones, teeth, gum, museum specimens and fossil insects) of different ages, albeit always less than one million years BP, and results can also be obtained from much older materials using palaeoproteomics. Nevertheless, new attempts to determine if ancient DNA (aDNA) is present in insects preserved in 40 000-year old sub-fossilised resin, the precursor of amber, have been unsuccessful or not well documented. Resin-embedded specimens are therefore regarded as unsuitable for genetic studies. However, we demonstrate here, for the first time, that although a labile molecule, DNA is still present in platypodine beetles (Coleoptera: Curculionidae) embedded in six-year-old and two-year-old resin pieces from *Hymenaea verrucosa* (Angiospermae: Fabaceae) collected in Madagascar. We describe an optimised method which meets all the requirements and precautions for aDNA experiments for our purpose: to explore the DNA preservation limits in resin. Our objective is far from starting an uncontrolled search for aDNA in amber as it was in the past, but to start resolving basic aspects from the DNA preservation in resin and search from the most modern samples to the ancient ones, step by step. We conclude that it is therefore possible to study genomics from resin-embedded organisms, although the time limits remain to be determined.

## Introduction

Deoxyribonucleic acid (DNA) contains the genetic information that allows all life forms to function, grow and reproduce; in addition, it is present in all cells and thus forms part of the tissues. Ideally, fossil—or ancient—DNA (aDNA) might serve as an alternative to morphological analysis and palaeoethology, which for centuries have served as the only tools available for scientists to determine the phylogenetic relationships between past organisms. Genomic sequences also provide insights into molecular evolutionary changes over time, clarify evolutionary relationships among taxa and yield information on mutualism rates [1, 2].

Amber is fossilised tree resin that may preserve deep time fauna in exceptionally clear morphological detail [3–5], and therefore appeared a promising material for preserving aDNA. It was hoped that DNA could easily be extracted from ancient soft tissue remains that were desiccated and macroscopically well preserved [6], because the complete and rapid engulfment of arthropods in resin and their hypothetically rapid fixation and dehydration—a type of mummification—promised to promote the preservation of DNA [7, 8]. Claims for DNA preserved in amber samples were rapidly made [9–12] in some cases with samples more than 125 million years old [13]. These studies overshadowed other less well-known studies on younger aDNA [14, 15]. However, amber claims are suspected of being the result of modern environmental DNA contamination because authentication procedures were not followed [16–22].

Natural resins are secreted from parenchymal cells in plants and trees and comprise complex mixtures of terpenoid compounds, including acids, alcohols and saccharides, some of which have preservative and antimicrobial properties [5, 23]. It has been observed that modern resin and amber both exhibit extensive chemical variations [24], which may influence preservation processes and therefore the quality of embedded organisms [25, 26]. Furthermore, DNA is a particularly labile macromolecule. In living cells, specific repair mechanisms act on damaged DNA, but these processes cease after cell death and DNA is naturally degraded [27]. Besides active cleavage of DNA by nucleases shortly after cell death [28, 29], long-term DNA degradation by hydrolytic processes (initiating double-strand breaks) or oxidative dinucleotide modification and depurination (removal of the guanine and adenine bases from the sugar-phosphate backbone) highly affect the stability of DNA molecules [16, 30–33]. Additionally, it seems that diagenetic events, including overburden pressure and heat generated through orogenesis over millions of years, affect amber permeability [34] and minimise the likelihood of DNA preservation in amber [21, 35].

The resin samples used in this study came from *Hymenaea verrucosa* Gaertner, 1791 [36] trees (Fabaceae), which produce copious amounts of resin that contains different types of sesquiterpene hydrocarbons and diterpenoid resin acids [23, 37]. Plants from this genus are the source plant of Miocene amber deposits in Mexico, the Dominican Republic, Peru and Ethiopia, and of sub-fossil resin deposits in different parts of the world [38]. Cenozoic amber from India and China was produced by some species of Angiospermae: Dipterocarpaceae [39, 40]. By contrast, the major Mesozoic amber deposits mainly derive from Coniferales [38]. The different plant group origin of the resin implies different sets of compounds in their chemical composition: monosaccharides, alcohols, aldehydes and esters [23]. The participation of esters in resin fossilisation (amberisation) and the biomolecules of the inclusions is still unknown [26]. Resin is a very complex (and not completely studied) preservation source with many variables simultaneously influencing preservation processes.

Distinguishing ancient DNA from recent contamination may be difficult while no strict correlation exists between DNA degradation and the age of the studied organism [41]. Although a comparison of modern and older mitochondrial DNA (mtDNA) showed significant differences in average fragment size, there is no direct correlation between age and fragmentation, and it is assumed that the main processes affecting DNA fragmentation occur rapidly after cell death [41, 42]. Heintzman et al. [43] found a time-dependent decrease in the concentration of amplifiable DNA in museum specimens. As mtDNA degrades more slowly than nuclear DNA (nuDNA), presumably due to additional protection of mtDNA by the double membranes of the mitochondrion, and is present in many more copies per cell, mtDNA may be more useful for aDNA studies [43–45]. Many different factors influence the state of preservation [3, 4]; therefore, the conditions under which individual specimens have been preserved are of decisive importance [20].

Attempts in various laboratories to repeat aDNA extraction from amber or from younger resin that will become amber have not been successful [21, 46, 47], which has raised further doubts about claims that aDNA was isolated from various fossilised insects in amber. Penney et al. [21] used next generation sequencing to analyse two Colombian sub-fossil resin bees, dated as less than 60 years and around 10 600 years BP, respectively. The young age of these resins means that they were not subjected to any extreme processes. Nevertheless, no convincing evidence was obtained for the preservation of endogenous DNA in either of the two studied sub-fossil resin inclusions. The clear conclusion of this study was that DNA is not preserved in insect inclusions in sub-fossil resin and amber [21]. After publication of these (and previous) results, the hypothesis that preserved DNA could be extracted and studied from animal remains in amber, or in younger resin, was totally discarded. However, these studies did provide negative evidence, demonstrating that DNA was not preserved in these samples.

Some success with DNA amplification has been reported with pinned museum and permafrost-preserved invertebrates [43, 48–51], and some of these samples were older than the oldest specimens analysed by Penney et al. [21]. These studies concluded that museum specimens could serve as a source of molecular information [48–50]. Of all recent publications, the sole hypothetical success with aDNA extraction from sub-fossil specimens embedded in resin was reported by Büsse et al. [51], who claimed to have successfully amplified aDNA from 61 species of 46 higher arthropod taxa from ca. 200-year-old dried museum specimens, as well as from two beetles embedded in sub-fossil resin. The two samples of ancient resin (*sensu* [52]) were analysed via accelerator mass spectrometry (standard-AMS) radiocarbon and dated to 790–700 years BP and 4030–3900 years BP. However, the positive results reported by Büsse et al. [51] should be viewed with caution as the publication does not contain a detailed discussion of the authentication procedures employed. The greatest problem with many studies of insect aDNA is their lack of reproducibility [47], a requirement for scientific evidence. Further limitations of studies of specimens embedded in resin include the implicit destruction of the samples and the small size and often uniqueness of the specimen analysed. Here, we discuss possible ways to solve these questions and limitations.

Fortunately, many studies conducted since 2005 have analysed aDNA using more specific methods, such as next generation sequencing, which not only yields massive amounts of sequencing data and amplification of highly degraded DNA, but also enables more efficient exclusion of modern contamination [53, 54]. Additionally, aDNA research has yielded promising results [e.g. 18, 54–57]; positive studies have been reported for aDNA from beetles in museum collections and permafrost [43, 49] and more recently, novel insights have been obtained into biomolecule preservation in amber samples [58] and other fossils [2, 59], including chewed birch pitch [60].

Here, we describe attempts to amplify DNA from ambrosia beetles embedded in six-year-old and two-year-old resins (modern resin, *sensu* [52]) from Madagascar collected *in situ* from the producing trees. Authentication and potential contamination with modern DNA were taken carefully into consideration. Our objective was to explore the potential limits of DNA preservation in resins and to develop a standardised protocol for DNA extraction from these samples, which could guarantee unambiguous and independent verification of fossil DNA following the authentication procedures for aDNA research, but applied to a more modern samples by the moment.

## Material and methods

### Samples

**Fig 1:**
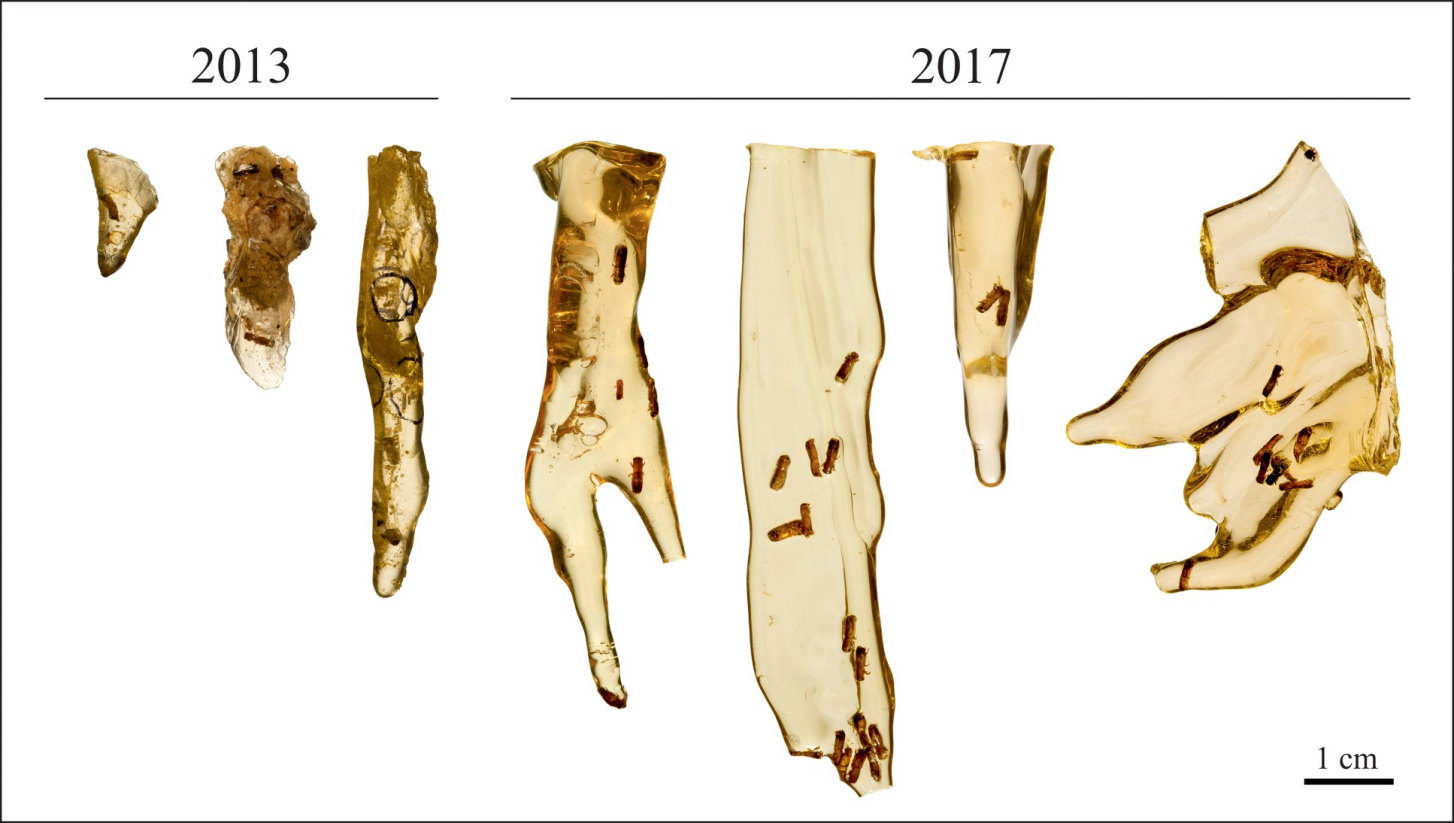
Resin samples from *Hymenaea* trees in Madagascar with embedded platypodine beetle specimens of the genus *Mitosoma*, sampled during fieldtrips in 2013 and 2017.

We used platypodine beetles of the genus *Mitosoma* Chapuis, 1865 [61] (Coleoptera: Curculionidae: Platypodinae) embedded in resin (Fig 1). *Mitosoma* is an endemic genus from Madagascar and is abundantly found embedded in resin drops from *Hymenaea verrucosa* (Angiospermae: Fabales: Fabaceae). We collected specimens in resins from a lowland forest close to the Pangalanes Canal, in Ambahy (Nosy Varika, Mananjary) (20º46’ S, 48º28’ W) and Andranotsara (Sambava) (14º37’ S, 050º11’ W) on the east coast of Madagascar. The Government of Madagascar authorised sampling (permit no. 160/13 /MEF/SG/DGF/DCB.SAP/SCB and no. 192/17/ MEEF/SG/DGF/DSAP/SCB.Re) and exportation (permit no. 186 N.EA10/MG13 and no. N. 249/17/MEEF/SG/DREEF-SAVA) of samples. The specimens embedded in resin were collected by X.D., E.P. and M.S.K. directly from *H*. *verrucosa* trees in Madagascar in October 2013 and September–October 2017, respectively. Resin samples were stored at room temperature until commencing the study in February 2019, which increased the risk of greater DNA degradation [27]. After the first experiments, all resin samples were stored at -20°C.

We used specimens of *Mitosoma lobatum* Schedl, 1961 [62], *M*. *excisum* Schaufuss, 1897 [63] and *M*. *obconiceps* Schedl, 1970 [64] as positive controls, collected in 2012 by B.J. when on a fieldtrip in Ranomafana National Park (Madagascar). Specimens were stored in >96% ethanol immediately after collection and preserved at -20°C prior to DNA extraction.

DNA extraction was performed at the Institute of Medical Microbiology, Immunology and Parasitology (University Clinic Bonn, Germany) from ten adult specimens in resin and four adult specimens in ethanol, all representing the same genus and body size, but different localities (see above) and ages (Table 1).

**Table 1:** Specimens of *Mitosoma* sp. used for DNA extraction and DNA concentration of the eluted DNA [ng/μl].

Year	Specimen	Body Part	DNA-Conc. [ng/μl]	260/280
2012	In ethanol	head	3.20 ng/μl	2.20
		thorax	6.90 ng/μl	1.70
		abdomen	27.30 ng/μl	2.04
	In ethanol	head	9.44 ng/μl	1.87
		thorax	15.67 ng/μl	2.20
		abdomen	66.24 ng/μl	2.00
	In ethanol	head	11.32 ng/μl	1.86
		abdomen	111.58 ng/μl	2.10
	In ethanol	head	13.86 ng/μl	1.99
		thorax	38.54 ng/μl	2.12
		abdomen	105.45 ng/μl	2.09
2013	Resin	whole body	6.67 ng/μl	1.64
	Resin	whole body	9.34 ng/μl	1.86
	Resin	whole body	17.88 ng/μl	1.93
2017	Resin	whole body	21.17 ng/μl	2.13
	Resin	whole body	33.95 ng/μl	2.07
	Resin	whole body	47.35 ng/μl	2.01
	Resin	whole body	48.90 ng/μl	2.06
	Resin + chloroform	whole body	2.90 ng/μl	1.87
	Resin + chloroform	whole body	3.60 ng/μl	1.63
	Resin + chloroform	whole body	3.95 ng/μl	1.67

DNA purity is depicted by the absorbance ratio at 260 nm and 280 nm (260/280).

Only resin pieces with two or more complete specimens as syninclusions were selected in order to use some of them for analysis and store the other(s) at -20ºC at the Senckenberg Research Institute (Frankfurt, Germany) under the collection numbers SMF Be 13578–13584. We hoped thereby to halt degradation of the hypothetically preserved biomolecules [27] and to preserve the specimen(s) for future controls and experiments. These specimens are available to other researchers/laboratories upon request, for suitable research projects aimed at determining reproducibility. Different specimens from other resin samples collected at the same time (three from 2013 and four from 2017) were treated as independent replicates (Table 1, Fig 1).

### Sample preparation and DNA extraction

All samples used in this study were entirely encased within *H*. *verrucosa* resin. The resins with insect inclusions were cut into small cubes with a sterile scalpel, leaving a few millimetres around the insect. The surfaces were sterilised with 0.1 M HCl and then washed in sterile water. All the following procedures were carried out under a laminar flow hood in a microbiology laboratory without any contact with entomological experiments. The resin cubes were ground with a micro-pestle in a 1.5 ml reaction tube to which 180 μl ATL-buffer with 20 μl proteinase K (Qiagen, Germany) was added. After incubation at 56°C for 72 h and occasional vortexing, the DNA was extracted using the DNeasy® Blood & Tissue kit (Qiagen, Germany), following the manufacturer’s instructions. DNA was eluted in DNase- and RNase free water and stored at -20°C until further experiments. DNA concentration was measured using the NanoDrop™ One/OneC microvolume-UV/VIS-spectrophotometer (Thermo Scientific, USA). DNA extraction from beetles in ethanol was performed using an identical procedure except for a reduced lysis step of 24 h. To test an alternative methodology some cubes were incubated in 5 ml 100% chloroform (AppliChem GmbH, Germany) for 3 days at 40°C to dissolve the resin. After the chloroform treatment the beetle was washed in ≥ 99% EtOH and further processed as described above. Precautions to eliminate contamination included regular disinfection of all surfaces and working materials with Freka®-NOL AF (Dr. Schumacher GmbH, Germany) and the use of dedicated protective clothing, equipment and reagents. In addition, prior to DNA extraction and amplification experiments, working materials and surfaces were cleaned with DNA-ExitusPlus™ IF (AppliChem GmbH, Germany) to avoid contamination with extrinsic DNA.

### Primers

Primers and protocols were selected from previously published studies on the weevil subfamily Platypodinae [65–67], or newly designed based on Platypodinae DNA sequences. The primer pairs S3690F and A4285R, targeting the D2–D3 domains of the large nuclear ribosomal subunit (28S), and S2442F with A3014R, targeting the 3’ end of the mitochondrial cytochrome oxidase I (COI), efficiently detected DNA from extant *Mitosoma* samples and were therefore selected for further experiments (Table 2). Two additional COI primers (Table 2; COIRes F and COIRes R2; COInew) were designed based on sequences from the resin-embedded beetles collected in 2013. These enabled amplification of a much smaller fragments than the standardised primers used in previous studies (160 base pairs (bp) vs. more than 600 bp) and were more suitable for new experiments with older (and theoretically more fragmented) material.

**Table 2:** Primer sequences for DNA extraction selected from the literature and two new sequences tested here.

Targeted Gene		Sequence	Fragment Size	Annealing Temperature	Reference
Large ribosomal subunit (*28S*)	28S	(S3690F) GAG AGT TMA ASA GTA CGT GAA AC	~ 800 bp	55°C	[66]
		(A4285R) CTG ACT TCG TCC TGA CCA GGC		55°C	[66]
Cytochrome oxidase I (*COI*)	COI (original primer pair)	(S2442F) CCA ACA GGA ATT AAA ATT TTT AGA TGA TTA GC	~ 600 bp	49°C	[67]
		(A3014R) TCC AAT GCA CTA ATC TGC CAT ATT A		49°C	[67]
	COInew (modified primer pair)	(COIRes F) CAG TAT TTG CTA TCT TAG CTG G	~ 160 bp	50°C	This work
		(COIRes R2) CGT GGT ATT CCT CTT AAA CC		50°C	This work

### Polymerase Chain Reaction (PCR) amplification

The PCR reaction mixture used to amplify genes of interest was composed of 12.5 μl One*Taq®* 2X Master Mix with Standard Buffer (New England Biolabs, Germany), 0.5 μl of each primer (10 μM) and 50 ng DNA, adding water to a final volume of 25 μl. Addition of 1 μl bovine serum albumin (BSA) (10 mg/ml) to the PCR reaction mixture improved efficiency and enabled a reduction in PCR cycles. In each PCR, a negative (sterile water) and a positive control (beetle DNA from specimens in ethanol) were included. PCR was performed in a Gradient LabCycler (SensoQuest, Germany) with the standard cycle program: initial denaturation step at 95°C for 5 min, followed by 30–50 cycles of denaturation at 95°C for 30 s, annealing at specific temperatures (Table 2) for 30 s and elongation at 68°C for 60 s, and a final elongation step at 68°C for 5 min. Further optimisation included different cycles and gradient PCRs to determine the annealing temperature. The PCR results were analysed by means of agarose gel electrophoresis (1.5%). The PCR products were purified using the GeneJet PCR Purification Kit or GeneJet Gel Extraction Kit (Thermo Scientific, USA), in case that fragments had to be extracted directly from the agarose gel, following the manufacturer’s instructions. Sanger sequencing was performed by Eurofins Genomics GmbH (Germany) with the amplification primers for 28S and COI (Table 2). Alignments between DNA sequences from beetles in EtOH and resin-embedded specimens to analyse the sequence data and investigate potential contamination with modern DNA were performed with Geneious R10 (https://www.geneious.com).

### Authentication of DNA sequences

We applied the following criteria to authenticate the amplified DNA sequences recovered from the beetles [41, 68]:

1. Negative controls, extraction blanks and PCR negative controls should be devoid of specific PCR amplification products.
2. Amplified sequences should be consistently and reproducibly obtained from the same extract(s) from one specimen and from different samples of the same species to guarantee reproducibility.
3. All procedures should be repeated three times in different laboratory rooms (in our case, at the Institute of Medical Microbiology, Immunology and Parasitology).
4. All PCR products should be controlled and analysed via sequencing for quality, assembly and specificity.

### Illustrations

General pictures of the resin samples were taken using a Nikon D3X with an AF-S Micro-NIKKOR 60 mm 1:2.8 G ED lens. Gel electrophoresis pictures were created using the FastGene FAS-Digi imaging system (NIPPON Genetics Europe). Detailed pictures of the beetle specimen were taken in the laboratory using a Keyence VHX1000 digital microscope under incident light (general body) and using a stereomicroscope in combination with a smartphone adapter. All figures were edited using CorelDraw-X8 software.

Original uncropped and unadjusted images underlying all gel results reported in this work may be found in the S1 Raw images.

### Statistics

Significant differences between DNA concentrations from the analysed samples were calculated by an unpaired t-test using GraphPad Prism version 5.00 for Windows (GraphPad Software, La Jolla, California, USA; www.graphpad.com).

## Results

**Fig 2:**
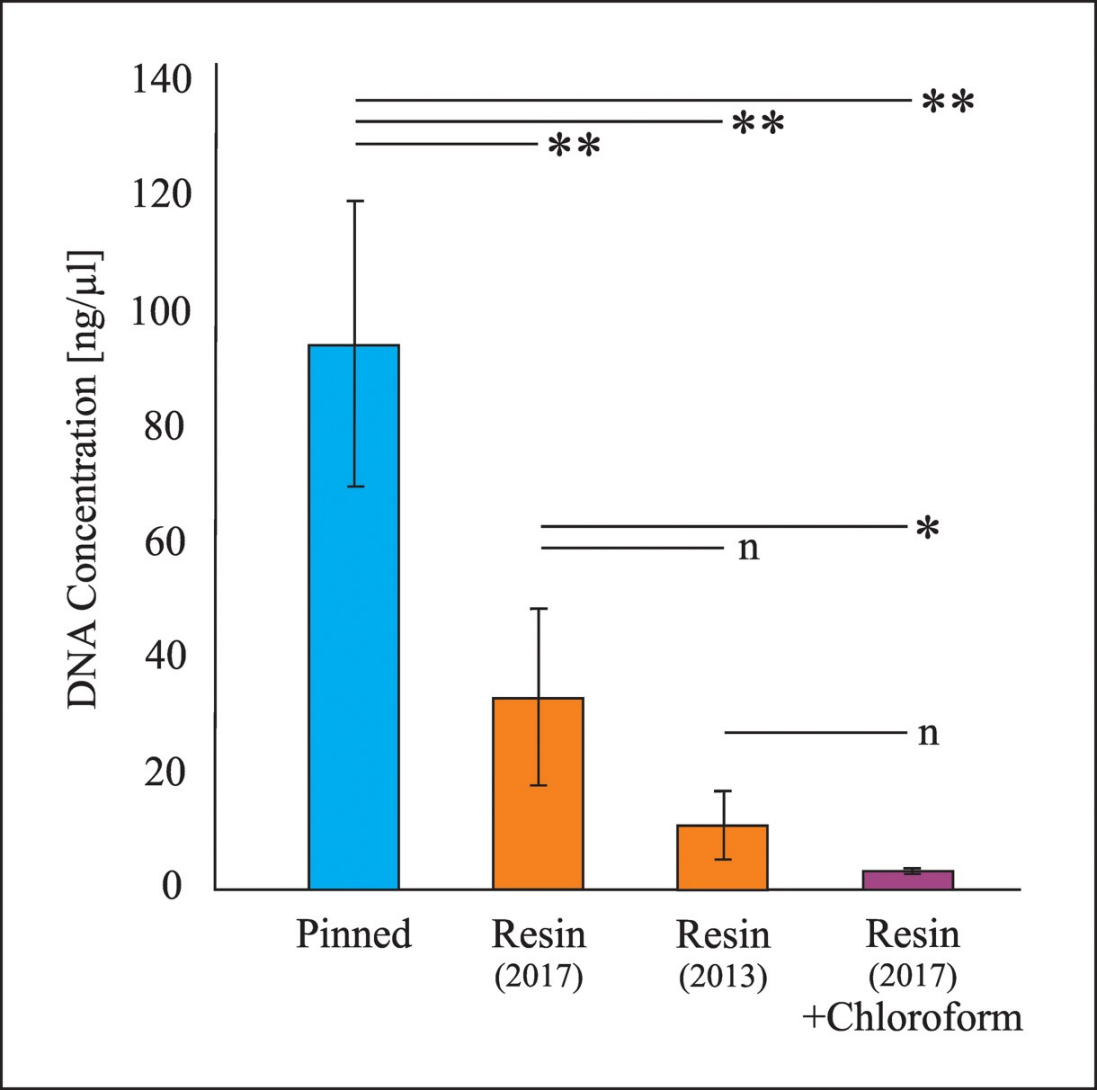
Mean DNA concentration in the different samples analysed. * = Significant differences P < 0.05; ** = Significant differences P <0.01; n = Non-significant differences.

Contrary to some previous experiments using insects in modern resin and sub-fossil resin [21, 47], we successfully amplified DNA sequences from beetles preserved in resins that were six and two years old (Fig 2).

### DNA extraction

DNA was extracted from beetle specimens of *Mitosoma* sp. and from specimens of the same genus preserved in resin of the same origin (*H*. *verrucosa*) but differing in age (2013 and 2017 fieldtrips) and collection site. DNA concentrations differed significantly between recent and resin-embedded beetles (Table 1). We observed a time-correlated decrease, albeit not significant, in the amount of DNA obtained from resin beetles (Table 1, Fig 2). The lowest DNA concentration was detected in resin beetles when the resin encasing the specimens had been dissolved in chloroform prior to DNA extraction (Table 1, Fig 2).

### PCR amplification

**Fig 3:**
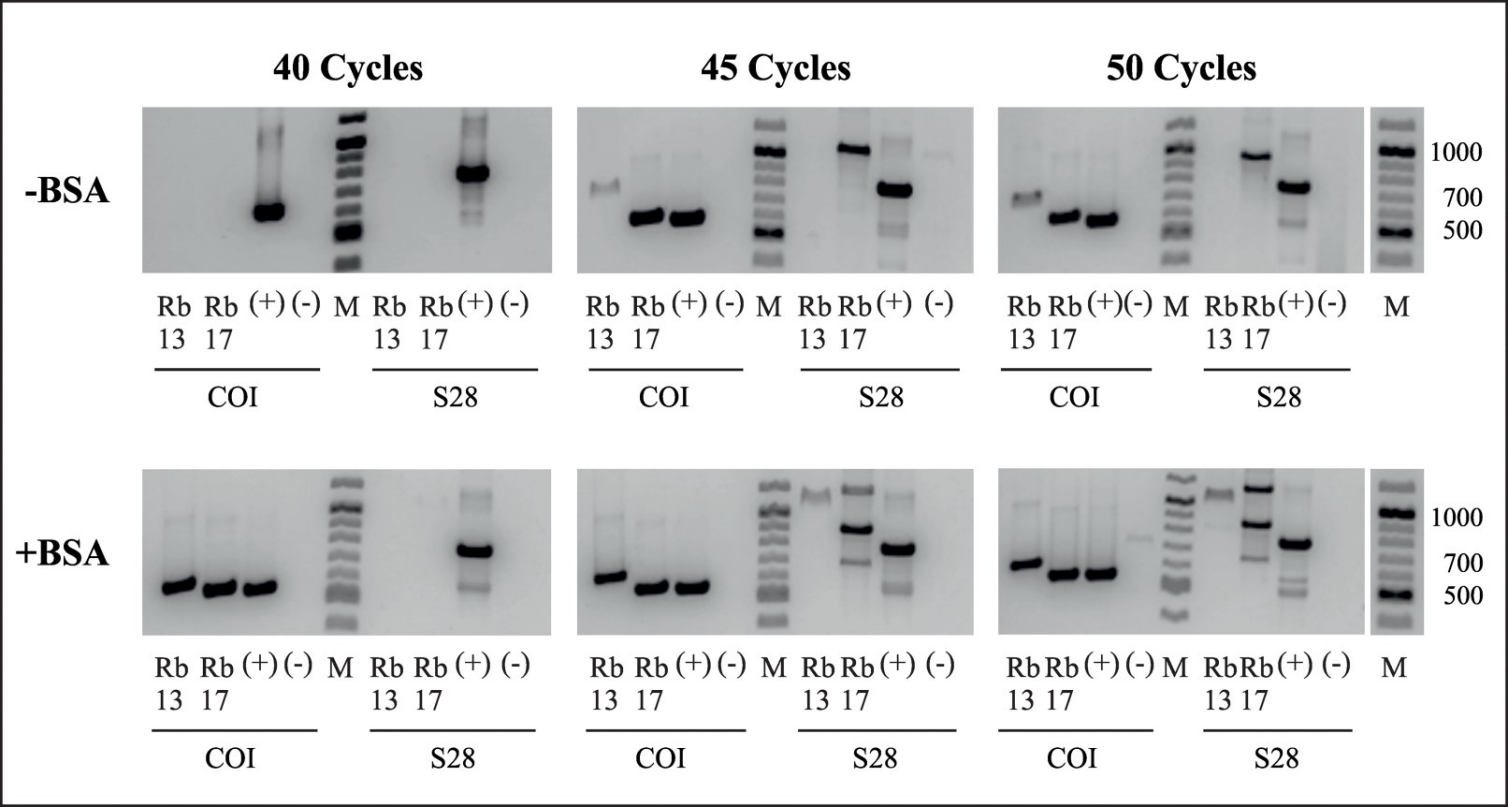
Gel electrophoresis of specific DNA fragments amplified with the original primer combinations COI and 28S with different numbers of cycles. Comparison of PCR products with and without the addition of BSA to prevent DNA polymerase inhibition. Rb13 = Resin beetle (collected in 2013); Rb17 = Resin beetle (collected in 2017); (+) = positive control, DNA from beetles in ethanol; (-) = negative control, DNase- & RNase-free water; M = 100 bp DNA ladder (New England Biolabs).

**Fig 4:**
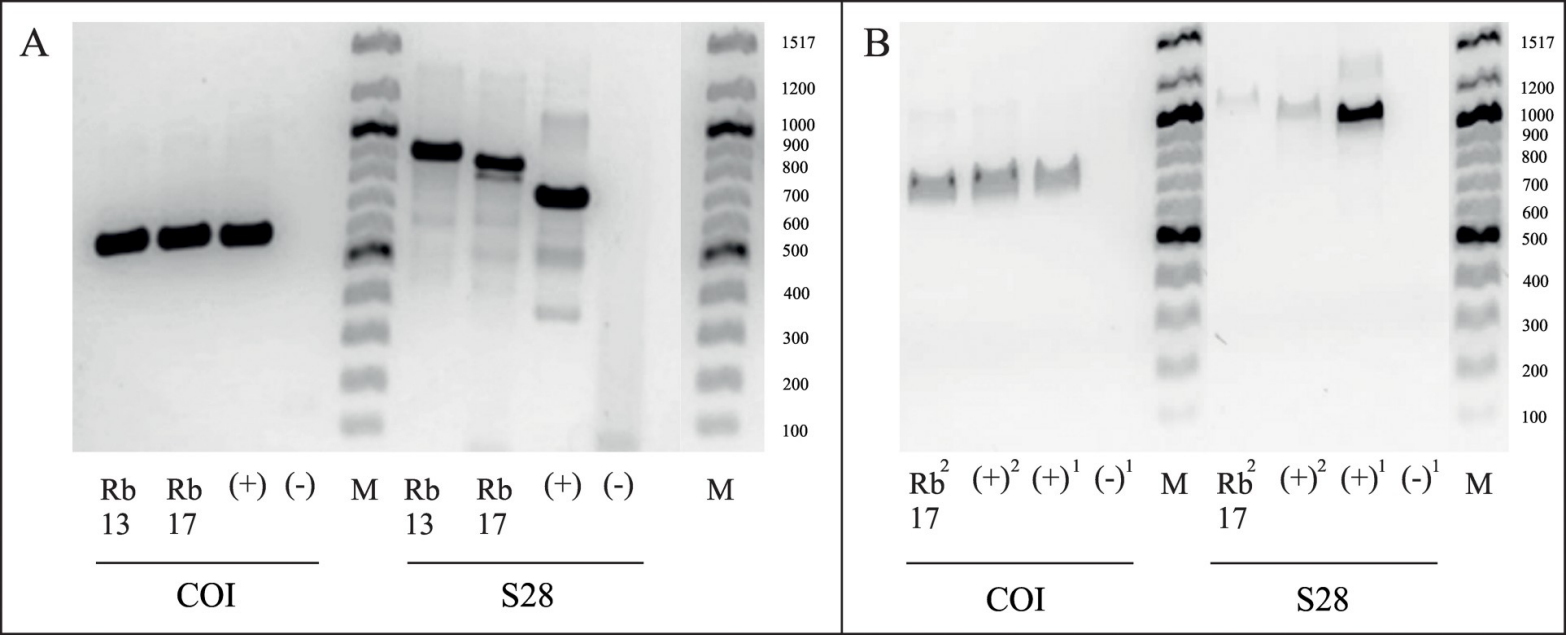
Gel electrophoresis of specific DNA fragments amplified with original primer combinations COI and 28S. **A**. Positive results with an increase in number of cycles to 50. Rb13 = Resin beetle (collected in 2013); Rb17 = Resin beetle (collected in 2017); (+) = positive control, DNA from beetle in ethanol; (-) = negative control, DNase- & RNase-free water; M = 100 bp DNA ladder (New England Biolabs). **B**. Two-step PCR with a PCR product purification step in between. Rb17^2^ = Resin beetle (collected in 2017) after two consecutive PCRs of 30 cycles each; (+)^1^ = positive control, DNA from beetle in ethanol after one PCR of 30 cycles; (+)^2^ = positive control, DNA from beetle in ethanol after two consecutive PCRs of 30 cycles each; (-)^1^ = negative control, DNase- & RNase-free water after one PCR of 30 cycles; M = 100 bp DNA ladder (New England Biolabs).

Initial attempts to amplify DNA from two- and six-year-old resin samples, using the PCR protocol normally employed with recent DNA, were unsuccessful, whereas amplification of recent samples (beetles in ethanol) was positive with the original primer pairs COI and 28S. As DNA extraction from the resin samples was performed with completely ground insect together with remaining resin portions, it was suspected that the resin components might inhibit the PCR. This was tested by the addition of different proportions of resin to beetle samples at different time points during DNA extraction. Neither addition of resin to the sample before DNA extraction nor mixing of extracted DNA from resin and recent samples (Table 3) supported the hypothesis of PCR inhibition by resin components. In further experiments, BSA was added to the PCR mixture to avoid any possible inhibition of the DNA polymerase (Fig 3). The blood plasma protein efficiently binds to inhibitors and thereby prevents a negative impact of these molecules on the DNA polymerase [69–71]. After that, two different approaches to amplify the specific DNA fragments were tested: a) two consecutive PCRs of 30 cycles each with a PCR product purification step in between, and b) an increase in the number of PCR cycles up to 50. Both strategies resulted in positive amplifications (Fig 4). Whereas the addition of BSA increased the amplification efficiency of the primer combination targeting the large fragment of COI and enabled successful amplification with a lower number of cycles (≥ 35 cycles), no product could be detected for 28S with less than 45 PCR cycles (Fig 3).

**Table 3:** Inhibition test performed with the primer combination COI for different proportions of DNA from beetles in ethanol and resin beetles.

Test	Proportion of DNA from resin beetles	Proportion of DNA from beetles in ethanol	DNA amplification result
1	1 μl (50 ng)	0 μl (0 ng)	Negative
2	0.8 μl (40 ng)	0.2 μl (10 ng)	Positive (weak)
3	0.5 μl (25 ng)	0.5 μl (25 ng)	Positive
4	0.2 μl (10 ng)	0.8 μl (40 ng)	Positive
5	0 μl (0 ng)	1 μl (50 ng)	Positive

Negative amplification was obtained when the DNA from beetles in ethanol was absent, weak positive result when the proportion was low and positive in the remainder of tests, even when resin remains were included.

### Control for contamination

Authentication procedures in aDNA studies are aimed at avoiding sample contamination with modern environmental DNA and detecting this should it occur. Therefore, each PCR product was purified and then sequenced by Sanger sequencing. To analyse the sequences several alignments were performed. Besides alignment of the sequences to Platypodinae sequences in the NCBI database BLAST to test for specificity, all products were checked for contamination with modern DNA. We especially examined the risk of inter-sample contamination from the positive control (beetles in ethanol) to the tested samples by multiple alignments of all sequences. Whereas the similarity between the sequences of insects extracted from resin in 2013 and 2017 was ≥ 97.4% with both primer combinations, a comparison between resin beetles and the sequence of beetles in ethanol yielded a similarity of only 73.7% for the original primer pair COI and 52.5% for primer combination 28S, respectively (Table 4), excluding any contamination with DNA from beetles in ethanol.

**Table 4:** Multiple alignments of all analyzed sequences and the similarity value for the different combinations.

Alignment	Primer-combination	Similarity [%]
Resin beetle (2013 + 2017)	original COI	97.4
Resin beetle (2013 + 2017)	28S	97.8
Resin beetle (2013 + 2017) alignment to beetles in ethanol	original COI	73.7
Resin beetle (2013 + 2017) alignment to beetles in ethanol	28S	52.5

## Discussion

### Contamination occurring during analysis

A major handicap in aDNA research, especially when using resin samples, is contamination with modern environmental DNA [16–20]. The selection of gene sequences for DNA amplification is a crucial step before working with genetically unknown organisms, as is the case of extinct organisms. Here, the closest living relatives often form the basis for identification of homologous sequences. In this study, primers were selected and specifically designed for beetles of the subfamily Platypodinae [66, 67], to prevent amplification of contaminating modern DNA. Furthermore, our experiments were performed in a microbiology laboratory experienced in molecular methods, but which had never worked with entomological DNA before.

To avoid contamination with modern DNA in the experiments presented here, several control procedures at different stages of the isolation and amplification processes were included in the experimental setup. Besides precautions for working with DNA, all PCR products were purified, sequenced and controlled for sequence specificity and inter-sample contamination. Relatively long fragments of the 28S ribosomal subunit (~ 800 bp) and the mitochondrial gene oxidase (~ 600 bp) (Table 1, Fig 2) were successfully amplified.

**Fig 5:**
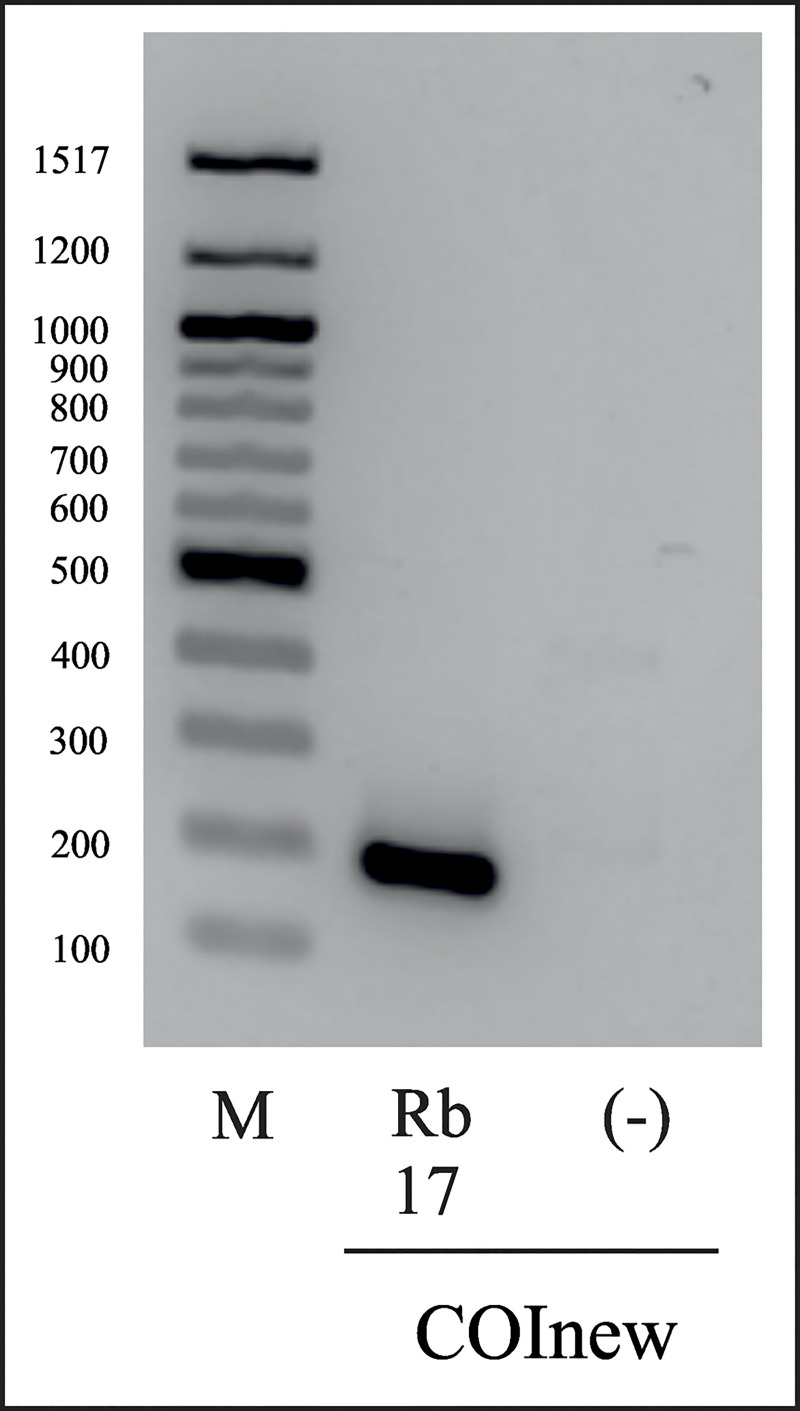
Gelelectrophoresis of the specific DNA fragments amplified with primer combination COInew. The primers were designed based on specific DNA sequences from resin-embedded beetles (collected in 2017) to amplify smaller DNA fragments. Rb17 = Resin beetle (collected in 2017); (-) = negative control, DNase- & RNase free water; M = 100 bp DNA ladder (New England Biolabs).

To take into account aDNA fragmentation due to decay, the primer used for amplification should encase small sequences. A basic premise is that primers should amplify fragments up to about 160 bp, because longer aDNA sequences become damaged and degraded and have generally proved impossible to amplify [18, 41, 68]. We designed primers for the amplification of small fragments (~ 160 bp) (Table 2; COInew) for future experiments. The new primers were tested successfully in first experiments with resin-embedded platypodine specimens from 2017 (Fig 5) and therefore, build a fundament for future experiments with aDNA using the same insects after confirming the results obtained by longer fragments.

### Reproducibility

Entomological studies can easily implement most recommended aDNA authentication procedures to increase the degree of reliability [18–20]. However, other criteria, such as reproducibility or independent replication, are difficult to meet with insect samples due to the small size and frequent uniqueness of the samples. The methodology entailed with samples embedded in resin renders the research even more difficult. The establishment of authentication procedures for aDNA from small samples requires further consideration [27]. Since aDNA isolation in amber insects requires the complete destruction of fossil material, which is obviously undesirable when dealing with rare and very old species stored in museum collections, a more feasible approach would be to analyse several individuals of the species of interest or individuals of any related species of a group [68]. A taxonomic group with relatively abundant representatives would be the perfect target for these experiments; when a specimen is truly unique, samples cannot be used for this purpose because of obvious methodological and ethical problems [27]. The problem of reproducibility is solved here by the selection of insect samples in resin that contain co-specific syninclusions (minimum 2), with a comparable degree of preservation. After cutting the resin sample, the remaining syninclusions were stored at -20°C to maintain their stability as far as possible and thereby preserve the DNA for future tests. Although they do exist, it is difficult to find similar syninclusions in older resins. For rare or unique finds, the approved protocol is to archive independent similar results in two independent laboratories [17, 68]. However, it is extremely challenging to achieve this when using insects from resin or amber deposits because it is difficult to independently replicate DNA extraction from the same fossil insect. In addition, resins of different origin should be treated as independent experiments because of the different nature of their chemical compounds.

Taking into account all the authentication procedures to increase the degree of reliability and avoid contamination in aDNA studies [17–20, 27, 68], we summarise here the optimal method that we employed, which we consider should become standard practice in taphonomic research on DNA preservation in modern and ancient resins:

1. Analyse the sample in a DNA laboratory that has not previously worked with organisms similar to the target specimens, preferably with previous experience in aDNA research.
2. Select a taxonomic group with abundant representatives as syninclusions in modern resin, sub-fossil resin or amber.
3. Include negative controls, extraction blanks and PCR negative controls.
4. Use specific primers for the target specimens instead of more general primers which amplify a broader range of genomes.
5. Include a sequencing analysis for quality, assembly and specificity control.
6. Repeat the experiment, ideally with similar specimens from the same sample. Close taxonomic groups from the same sample and corroboration with a sequencing process may be sufficient to demonstrate reliability and discard contamination.
7. Include a detailed list of the protocols used in the experiments with a clear discussion of the authentication procedures employed.

### The use of chloroform in DNA extraction from specimens embedded in resin

The dissolution of resin, sub-fossil resin and amber in chloroform is a typical procedure to release the embedded insects for further analysis [21, 72]. Penney et al. [21] were unable to detect any insect DNA via next generation sequencing after the use of chloroform to dissolve the resin, and thus concluded that DNA was absent. Chloroform:phenol mixtures are frequently used for DNA extraction [73–75] and one might assume that the difference in age between the youngest sample analysed in Penney et al. [21] and the oldest one in this study (6 years vs. 60 years) could be a key factor in our successful amplification. Surprisingly, we found that the use of chloroform to dissolve the resin surrounding the specimens compromised DNA concentration (Table 1, Fig 2). During work with DNA samples, the use of 70% ethanol also poses a problem as DNA precipitates at this ethanol concentration [47]. Therefore, only ≥ 80% ethanol should be used to sterilise resin surfaces. Büsse et al. [51] reported the amplification of DNA sequences from specimens preserved inside sub-fossil resin samples, but the lack of information about the methodology used with the specimens embedded in resin precludes comparisons. Further experiments are required to obtain more strongly supported conclusions about the effects of chloroform and ethanol on DNA extraction from resin-embedded material.

### Dehydration of specimens

The life-like preservation of many amber inclusions is accompanied by preservation of ultrastructural details such as muscle fibres [76], mitochondria [77], brain tissue [3], and internal genitalia [78]. After the death of an organism, enzymes such as DNases rapidly degrade the DNA, but some repair mechanisms, if active, can revert this process. Under rare circumstances, tissue rapidly desiccates after death [8, 79] and DNA is absorbed by a mineral matrix [80]; in addition, rapid burial may change surrounding conditions, significantly reducing or completely inhibiting enzymatic and microbial degradation [81, 82]. On such occasions, slow but still manifest chemical processes start to affect the DNA [18, 83, 84]. Cano [85] stated that sugars such as arabinose, galactose and sucrose, which are components of natural resins, increase osmotic pressure on cells and thereby draw out the water, which results in tissue dehydration and inhibition of biochemical reactions. Additionally, alcohols and terpenes may act as fixatives to preserve tissues.

**Fig 6:**
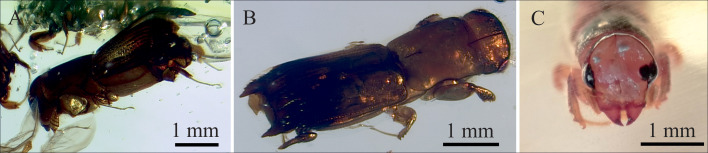
Different specimens of *Mitosoma* sp. preserved in resin from *Hymenaea verrucosa* collected in the Sambava region (Madagascar) in 2017. **A**. Lateral view of a complete specimen. **B**. Dorsal view of a complete specimen. **C**. Photomicrograph of the sectioned head of one *Mitosoma* beetle in resin, showing the still fleshy internal tissue after two years inside the resin.

Dehydration and preservation in resin or permafrost are the three modes of preservation that retain more life-like organisms than any other kind of fossil, maintaining tissues over time [3]. While promising results have been obtained from permafrost-preserved invertebrates [43], none of the claims of DNA isolated from million-year-old amber samples could be independently replicated [21, 47]. In a recent study, amplified fossil genomes were extracted from birch pitch [60], another organic plant substance like resin, but obtained by heating birch bark. Supposedly, volatile compounds cause thorough and rapid dehydration and fixation of tissue once the trapped organism comes into contact with the resin [3]. This contrasts with our observations of resin-embedded *Mitosoma* sp. specimens from Madagascar (Fig 6). Two-year-old ground specimens still contained apparently flesh-like tissue without any indication of dehydration or shrinkage (Fig 6C). Therefore, hypothetical dehydration in the resin is not as rapid as was thought and must be regarded as the result of a process lasting for several years. Although the mono- and sesquiterpenoid volatile compounds in resins are known to have antimicrobial and enzyme inhibiting characteristics [23, 86, 87], it is possible that the di- and triterpenoid non-volatile compounds, some of which are also antimicrobial, are crucial to stabilise tissue over longer timescales. The lack of preservation of other molecules, such as chitin and lignin, which are more time-resistant than DNA, in Miocene amber-embedded insects and plants [88] has also been used to argue against the preservation of DNA in these particular fossils [18]. However, ß-1,3 and ß-1,4-linked polysaccharides, and specifically N-acetylglucosamine residues from chitin, have been detected in fungal mycelia in resinicolous fungi from Spanish amber [89]. One explanation for the better preservation of aDNA in bones than in resins may be hydroxyapatite. This mineral, which predominates in bone, is known for its very strong binding affinity to DNA [15, 80, 90]. Our observations indicate that there is still no satisfactory explanation for preservation in amber, but imply that water is available in the system for longer than previously thought, which has a negative effect on DNA stability in specimens embedded in resin.

### Future approach to determine the time limits of DNA preservation in resin specimens

There are broad areas where further progress in studying aDNA from resin-embedded insects can be expected. Decay experiments provide a useful means to investigate variations in the preservation quality of different resin types [26] and offer a promising subject for future analysis. The preservation of inner structures of insects was highly compromised after only a few months embedded in different kinds of resin [26], but to the best of our knowledge, DNA preservation has never been tested. A comparison of DNA preservation in dead specimens of varying ages (such as those from museums and collections) [91], or specimens fixed in paraffin or embedded epoxy resin from less than one hundred years ago, would be highly interesting. Based on the positive results from this study, future experiments could investigate the limits of the DNA preservation in resin embedded specimens in short-time intervals. For future approaches with samples highly sensitive to contaminations, it will be required further adjustments of the methodology. For instance, the DNA concentration was measured via a microvolume-UV/VIS-spectrophotometer in this study, which lacks specificity, being unable to distinguish between DNA (single- and double-stranded), RNA and nucleotides, and is prone to contamination. Therefore, we recommend to use a fluorometer, which is highly accurate detecting fluorescent nucleic acids. Additionally, DNA of older resin embedded specimens should be analysed with more sensitive next generation sequencing technologies such as shotgun sequencing. This method is more specific, enabling distinction between modern contaminations and ancient DNA and provides further possibilities, e.g. analysis of fragmentation or degradation patterns. Proteins are thought to be more stable than DNA [92, 93] and seem to provide similar phylogenetic information [2]. Some experiments detected biomolecules such as chitin-protein complexes in insect cuticle from sub-fossil resin but failed to do so when Dominican amber samples were tested [88], while other more recent studies found amino acids from fossil feathers in amber from almost 100 million years BP [58]. Therefore, proteins may be a promising target for the study of deep time specimens [94]. However, if the objective is DNA, it may be more effective to amplify mitochondrial DNA since this is present in higher copy numbers than nuclear DNA, rendering it a more promising candidate for genetic analyses of aDNA [43–45].

The experiments presented here were performed using the commercial DNeasy® Blood & Tissue Kit (Qiagen, Germany), which was recommended for the extraction of beetle DNA [65, 66]. Other studies have also tested DNeasy® extraction systems, which are based on the binding of DNA to silica membranes, for other types of sample, such as bones or other insects [69, 95]. As a beetle embedded in resin is a mixture of two sample types, tissue (insect) and environmental (resin), selection of the most suitable extraction kit must be carefully assessed. Environmental kits offer the advantage of additional steps to avoid PCR inhibitors [96–98], whereas tissue kits are specifically designed for DNA extraction from tissue samples. Other aspects such as choice of DNA polymerase can significantly affect PCR efficiency [69], and should be further investigated to identify the ideal DNA extraction and PCR amplification protocol.

The interaction between a fossil sample and its environment following death of the specimen determines the preservation state of the biomolecules; age *per se* is not an absolute indicator of quantity or quality, at least over a longer timescale [18]. Storage conditions also play a crucial role in preservation, and dry, cold conditions are best for DNA preservation [27]. Since each case is unique, attending to our results, no biological sample should be disregarded as a potential source for recovery [15].

## Conclusions

The existence of aDNA in amber specimens is dependent on the possibility that the resin provides a protected environment for DNA preservation (both protective, through encapsulated conditions, and chemically favourable). In previous studies, no aDNA could be amplified either in amber or in specimens embedded in resin, suggesting that the protective and preservative environment in resin prevents tissue but not DNA degradation. However, modern techniques are more powerful and more sensitive. Recently, Büsse et al. [51] reported successful amplification of aDNA from sub-fossil specimens, but limited information was given about extraction methods, the exclusion of contamination or aspects of reproducibility; therefore, the positive results obtained in that study should be viewed with caution. By contrast, the methodology discussed here is intended for future use as a guide and base for studies of specimens embedded in resin. In future investigations next generation sequencing should be included into the methodology as this technique provides more possibilities when working with aDNA. Our study was designed to clarify fundamental aspects about DNA preservation in resin-embedded insects, including an evident experimental support, that was absent until now. Our positive amplification demonstrates that resin inclusions can also be a resource to explore in aDNA studies, albeit with caution. The risk of contamination demands the design of suitable authentication procedures and the possibility to re-evaluate the inclusion of further analysis once the method is established. Subjects for future studies include the time limits for aDNA detection in resins, the preservation state and assessment of possible taxonomic bias (both entomological and plants) in long-term DNA integrity. With this in mind, a new research project has been launched, moving from newer to older resin samples in order to determine the time limits of DNA preservation in resins.

## Supporting information

S1 Raw images (PDF)
