# Supplementary material for: DNA from resin-embedded organisms: Past, present and future
Source: PLoS One. 2020 Sep 28;15(9):e0239521. doi: 10.1371/journal.pone.0239521 (PMC7521698; doi:10.1371/journal.pone.0239521)
Supplement: S1 Raw images — (PDF) [file pone.0239521.s001.pdf]

Fig. 3, pictures were taken with FastGene FAS-Digi (NIPPON Genetics Europe). As molecular marker 100 bp ladder (New Englang Biolabs) was used. The color was changed to Black and White and inverted.

Figure 3. Gelelectrophoresis of the specific DNA fragments amplified with primer combinations COI1 and S28 with different amounts of cycles. Comparison of the products of PCR reactions with and without the addition of BSA to prevent inhibition of the DNA polymerase. Rb13 = Resin beetle (collected in 2013); Rb17 = Resin beetle (collected in 2017); (+) = positive control, DNA from pinned beetle; (-) = negative control, DNase- & RNase free water; M = 100 bp DNA ladder (New England Biolabs).

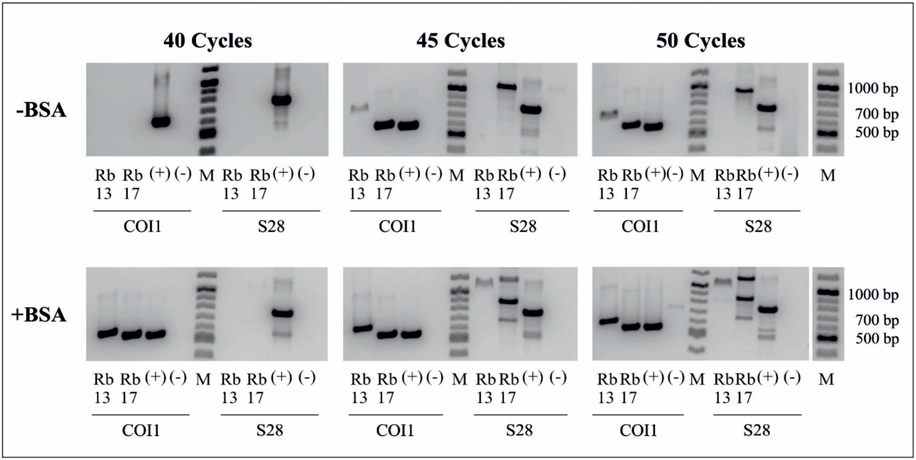

# -BSA / 40 Cycles

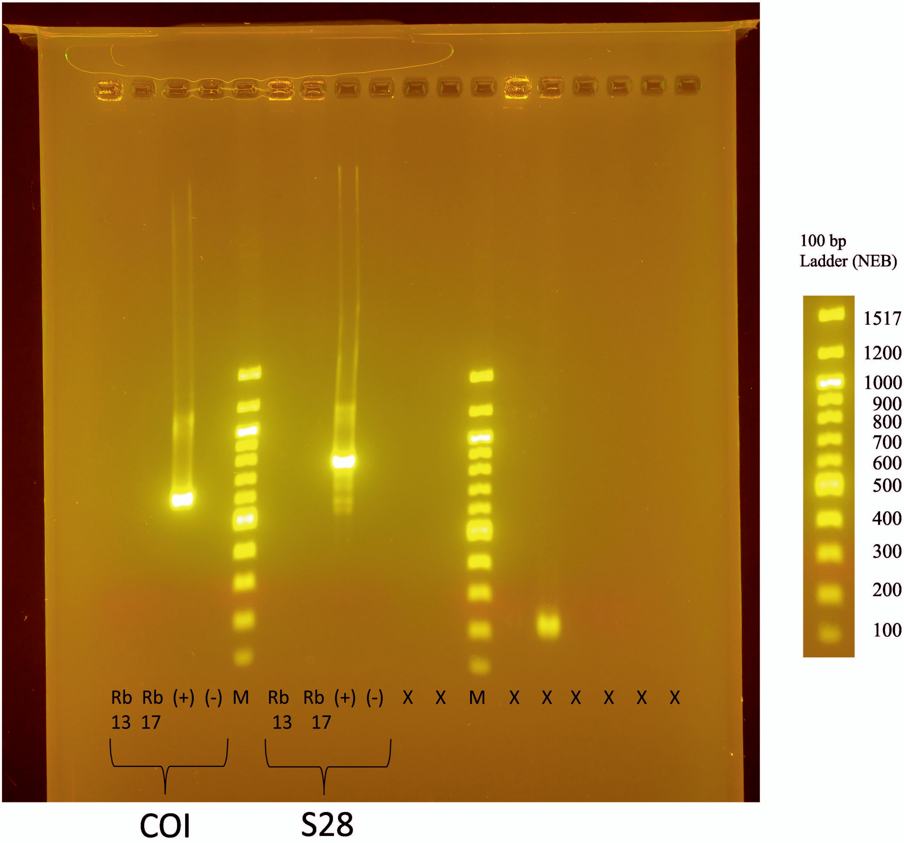

-BSA / 45 Cycles

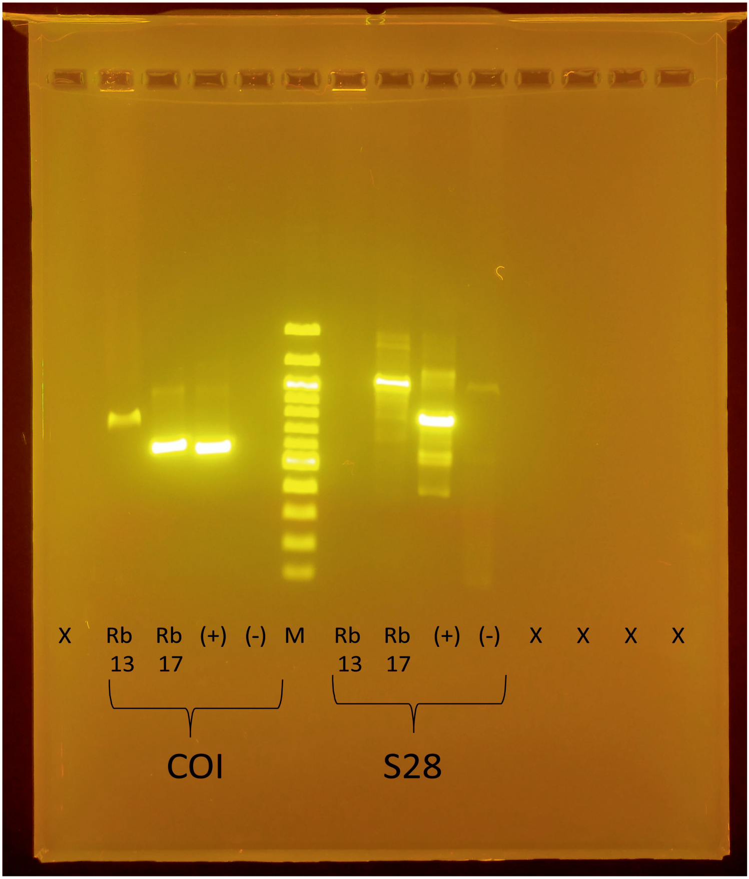


100 bp Ladder (NEB)

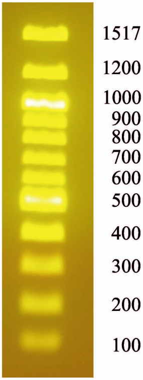

-BSA / 50 Cycles

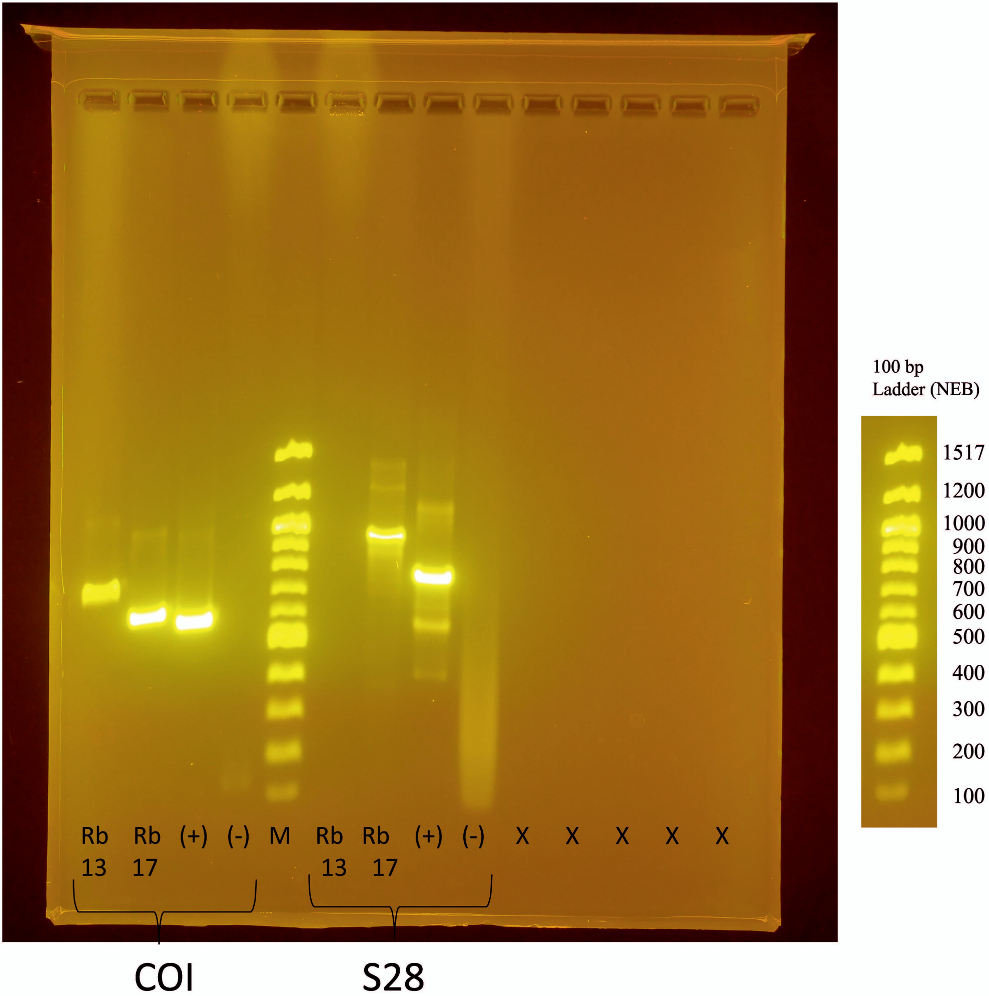

+BSA / 40 Cycles

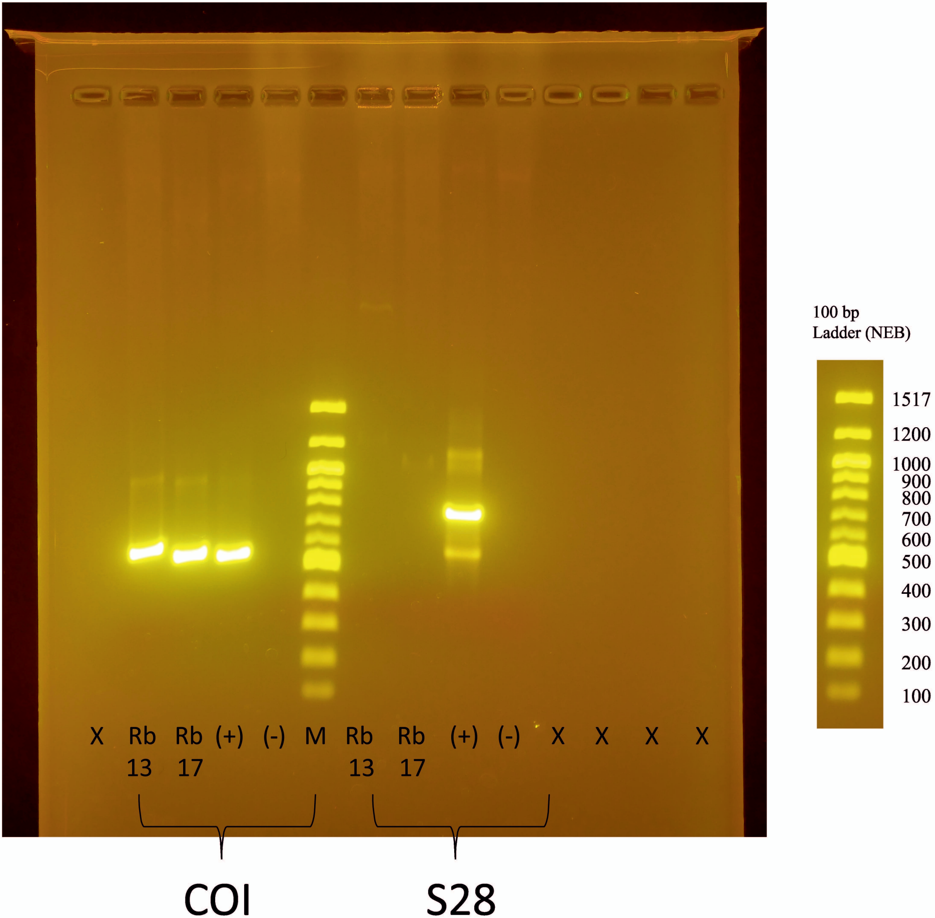

+BSA / 45 Cycles

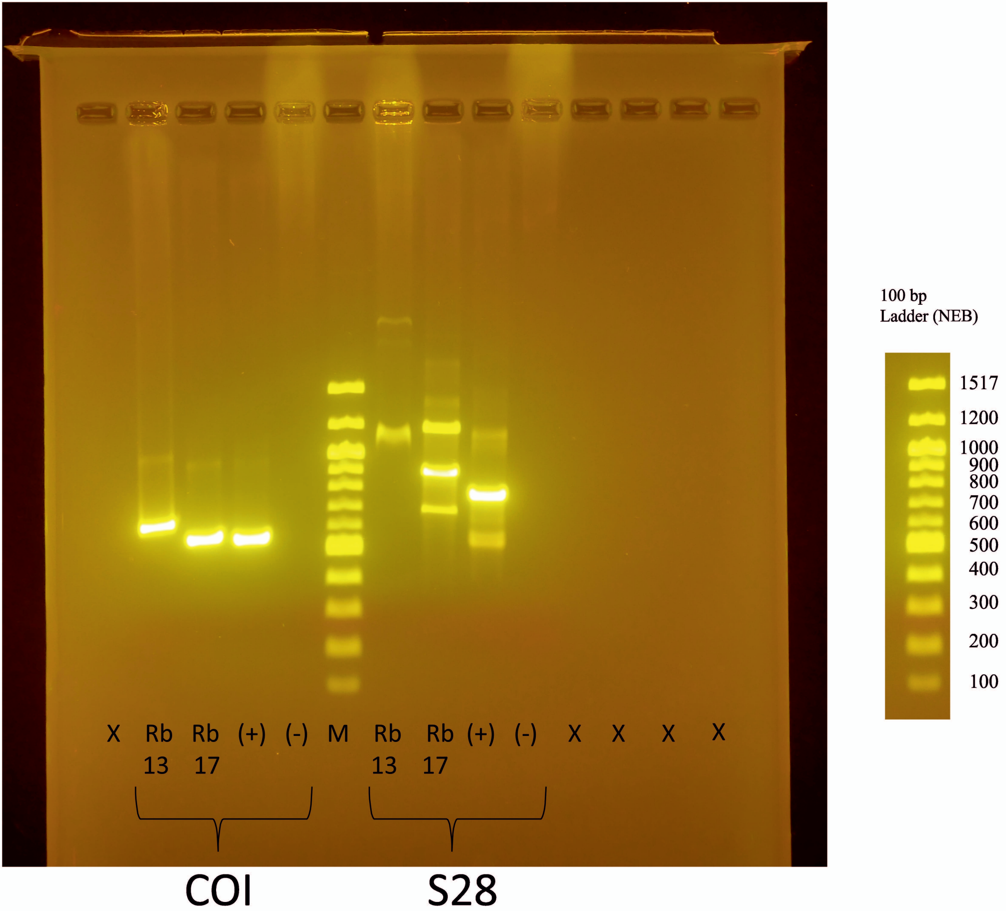

+BSA / 50 Cycles

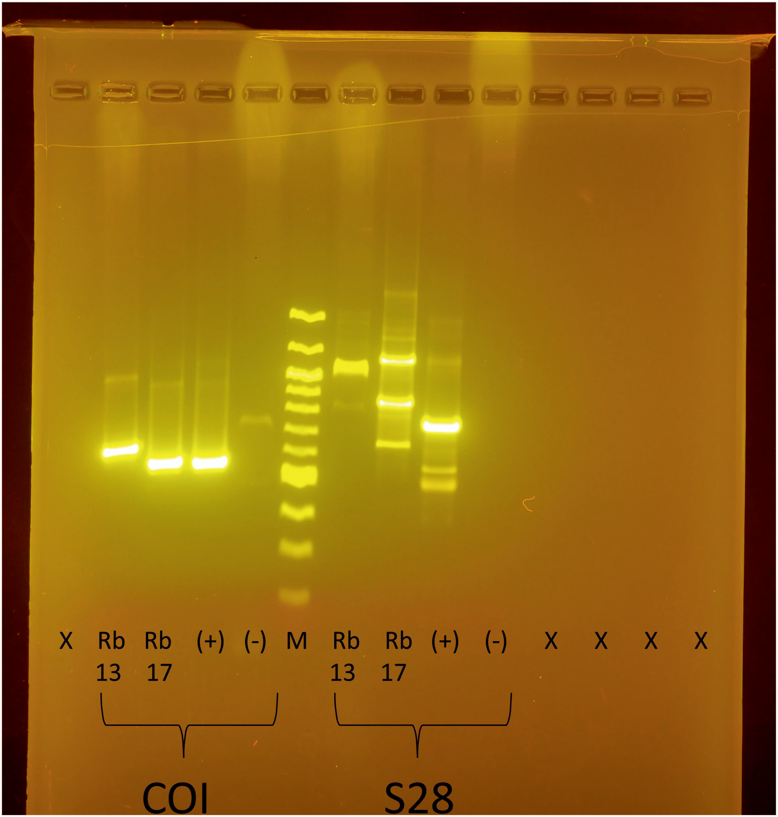


100 bp
Ladder (NEB)

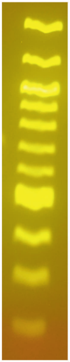

Fig. 4, pictures were taken with FastGene FAS-Digi (NIPPON Genetics Europe). As molecular marker 100 bp ladder (New Englang Biolabs) was used. The color was changed to Black and White and inverted.

Figure 4. Gelelectrophoresis of the specific DNA fragments amplified with primer combinations COI1 and S28. A. Positive results with an increase of amounts of cycles to 50. Rb13 = Resin beetle (collected in 2013); Rb17 = Resin beetle (collected in 2017); (+) = positive control, DNA from pinned beetle; (-) = negative control, DNase- & Rnase free water; M = 100 bp DNA ladder (New England Biolabs). B. Two-step PCR with a purification step of the PCR products in between. Rb172= Resin beetle (collected in 2017) after two consecutive PCR with 30 cycles each; (+)1 = positive control, DNA from pinned beetle after one PCR with 30 cycles; (+)2 = positive control, DNA from pinned beetle after two consecutive PCR with 30 cycles each; (-)1 = negative control, DNase- & RNase free water after one PCR with 30 cycles; M = 100 bp DNA ladder (New England Biolabs).

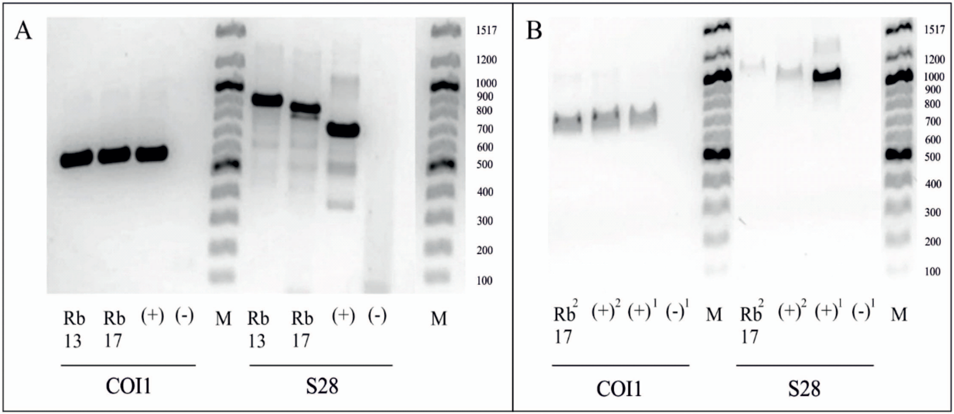

A. 50 Cycles

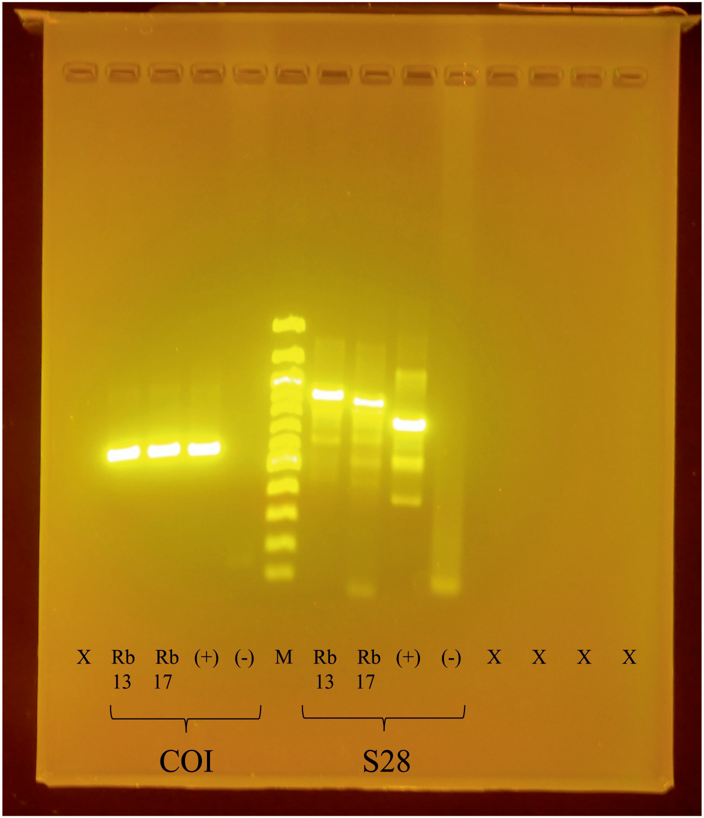
X
Rb 13
Rb 17
(+)
(-)
M
Rb 13
Rb 17
(+)
(-)
X
X
X
X
COI
S28

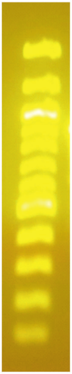
100 bp Ladder (NEB)
1517
1200
1000
900
800
700
600
500
400
300
200
100

B. 2-Step-PCR

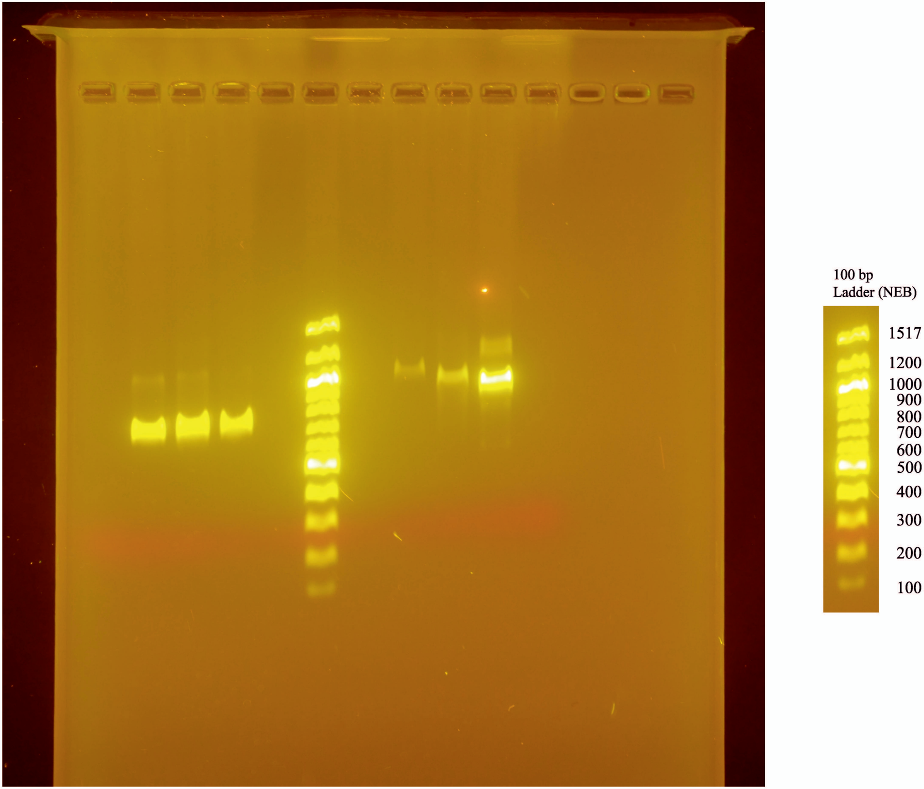


X Rb² 17 (+)² (+)¹ (-)¹ M X Rb² 17 (+)² (+)¹ (-)¹ X X X

COI S28

Fig. 5, pictures were taken with FastGene FAS-Digi (NIPPON Genetics Europe). As molecular marker 100 bp ladder (New Englang Biolabs) was used. The color was changed to Black and White and inverted.

Figure 5. Gelelectrophoresis of the specific DNA fragments amplified with primer combination COInew. The primers were designed based on specific DNA sequences from resin entombed beetles (collected in 2017) to amplify smaller DNA fragments. Rb17 = Resin beetle (collected in 2017); (-) = negative control, DNase- & RNase free water; M = 100 bp DNA ladder (New England Biolabs).

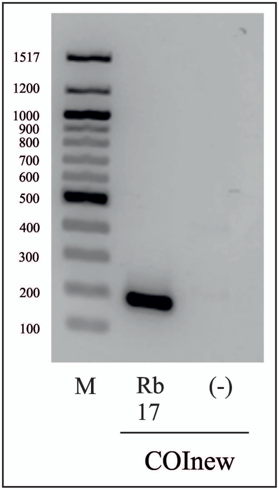

Fig 5

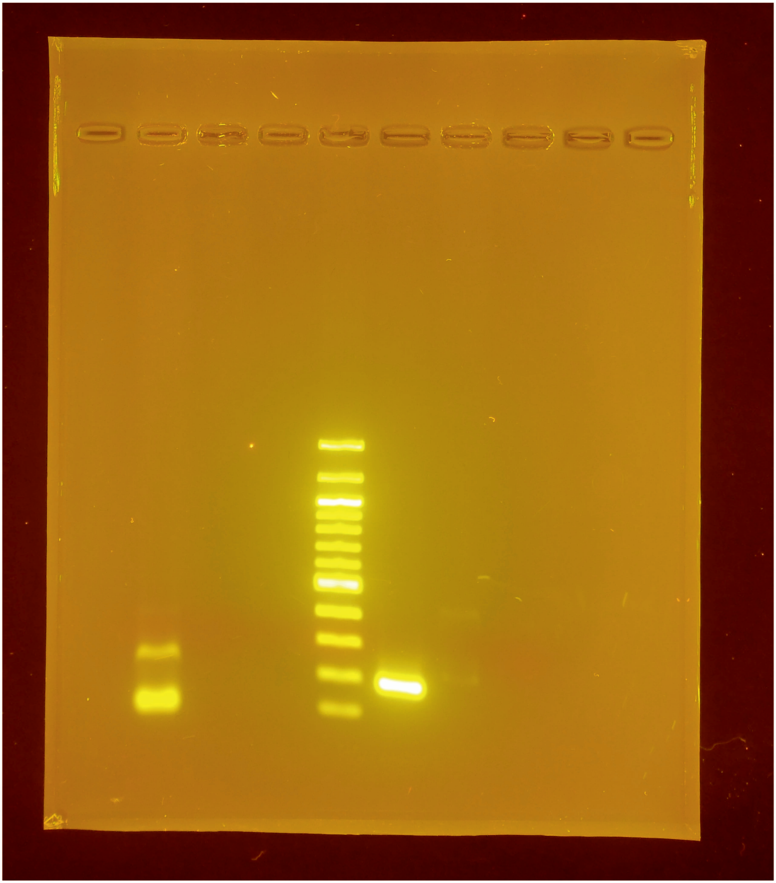

X X X X M Rb 17 (-) X X X

COInew

100 bp Ladder (NEB)

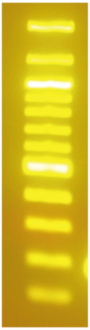

1517
1200
1000
900
800
700
600
500
400
300
200
100
